# Sex-specific associations of physical frailty with cardiac structure and function: a cross-sectional study based on UK Biobank

**DOI:** 10.1186/s12872-026-05737-5

**Published:** 2026-04-09

**Authors:** Gejing Liu, Shunlin Guo

**Affiliations:** 1 The First Hospital (First Clinical Medical School) of Lanzhou University, Lanzhou, Gansu 730000 China; 2 Department of Geriatrics, The First Hospital of Lanzhou University, Lanzhou, Gansu 730000 China; 3 Department of Radiology, The First Hospital of Lanzhou University, No. 1 Donggang West Road, Lanzhou, Gansu 730000 China

**Keywords:** Physical frailty, Cardiac magnetic resonance, Functional performance, Sex differences

## Abstract

**Background:**

Frailty, characterized by diminished physiological reserves and heightened vulnerability to adverse health outcomes, is a robust predictor of cardiovascular morbidity and mortality that extends beyond chronological age. Preclinical studies in aging mouse models have demonstrated that frailty—rather than age alone—drives adverse cardiac remodeling with distinct sex-specific patterns: ventricular dysfunction increases with frailty in males only, whereas atrial dysfunction affects both sexes. Whether these sex-dimorphic patterns translate to humans remains unknown. Epidemiologic data further highlight sex-related heterogeneity: men with frailty exhibit higher all-cause mortality, whereas frailty shows stronger associations with cardiovascular mortality in women, and heart failure with preserved ejection fraction (HFpEF) is approximately twice as prevalent in women. These observations raise the hypothesis that frailty associates with distinct cardiac phenotypes in men versus women.

**Methods:**

This cross-sectional study included 48,993 UK Biobank participants (24,946 women, 24047 men) who underwent Cardiac Magnetic Resonance (CMR). The Fried phenotype categorized physical frailty as Robust, Pre-frail, or Frail. Multivariate linear regression (adjusted for comorbidities and lifestyle) was used to evaluate the associations between frailty severity and CMR-derived parameters, stratified by sex.

**Results:**

Frailty was negatively associated with biventricular and atrial volume indices, left ventricular mass index, and left ventricular wall thickness in both sexes (all *p* < 0.01). Sex-frailty interaction analyses revealed that men exhibited greater sensitivity of ventricular structure to frailty: significant interactions were observed for left ventricular mass index (β=−1.474, *p* = 0.002) and left ventricular end-diastolic volume index (β=−1.455, *p* < 0.001), indicating steeper declines in men compared with women; a similar pattern was observed for right ventricular end-diastolic volume index (interaction β=−1.661, *p* < 0.001). Among functional parameters, only left ventricular ejection fraction showed a significant interaction (β = -0.007, *p* = 0.015), reflecting a compensatory pattern in men of preserving ejection fraction at the cost of structural remodeling. Women exhibited functional impairment, characterized by smaller left atrial ejection fraction (β=−0.797, *p* = 0.002) and decreased left ventricular global longitudinal strain negativity (β = 0.179, *p* = 0.028), but there are no significant sex-frailty interactions (*p* > 0.05).

**Conclusions:**

Physical frailty demonstrates sex differentiated associations with cardiac magnetic resonance phenotypes. Men exhibit predominant structural changes, with approximately 50% greater absolute reductions in cardiac volumes and mass per unit increase in frailty severity (e.g., LVMi reduction in frail men was 1.52 times that in frail women); women exhibit predominant functional changes, including left atrial dysfunction and reduced longitudinal strain. The sex differences in CMR parameters suggest that clinical monitoring strategies should consider both sexes. These findings highlight the importance of sex-specific approaches to assessing and managing cardiovascular risk in frail individuals.

**Supplementary Information:**

The online version contains supplementary material available at 10.1186/s12872-026-05737-5.

## Introduction

Frailty is a clinically identifiable state characterized by diminished physiological reserves and heightened vulnerability to a broad spectrum of adverse health outcomes [1]. Frailty has become more common due to the aging population [2]. Its prevalence increases markedly with age. While intrinsically linked to aging, frailty constitutes a pathophysiological condition that extends beyond chronological age and serves as a robust predictor of adverse clinical outcomes [3]. Frailty is an independent risk factor for mortality and is associated with an elevated risk of adverse cardiovascular events. These conditions include heart failure, coronary artery disease, and arrhythmias [4–7]. Notably, recent preclinical work in aging mouse models has demonstrated that frailty—rather than age alone—drives adverse cardiac remodeling, with distinct sex-specific patterns: ventricular dysfunction increases with frailty in males only, whereas atrial dysfunction affects both sexes [8]. These findings provide crucial mechanistic insights but require validation in human populations to establish clinical relevance.

Observational studies using echocardiography have reported an association between frailty and abnormalities in cardiac structure and function [9], including left ventricular diastolic dysfunction ( higher E/e′), concentric remodeling or LV hypertrophy [10], a less-negative LV global longitudinal strain (LVGLS) [9], and impaired left atrial conduit function [11]. However, echocardiography has limitations in quantifying myocardial tissue characteristics and precise chamber volumes. Cardiac magnetic resonance (CMR) represents the non-invasive reference standard for cardiac morphological and functional assessment, offering superior reproducibility and enabling precise quantification of chamber size, mass, pericardial adiposity, and tissue characterization. To date, CMR-based evidence on frailty remains scarce: only one study has examined the association between frailty and myocardial fibrosis using CMR, reporting no significant associations with atrial volumes or LV mass [12]. Moreover, whether the sex-specific cardiac remodeling patterns observed in preclinical models translate to humans remains unknown.

Epidemiologic data indicate sex-related heterogeneity across the frailty spectrum: men with frailty exhibit higher all-cause mortality, whereas the association between frailty and cardiovascular mortality appears stronger in women; Heart failure with preserved ejection fraction (HFpEF), a condition tightly linked to frailty, is approximately twice as prevalent in women as in men [13–15]. This discrepancy highlights the potential for sex-specific mechanisms underlying the relationship between frailty and cardiovascular disease. These observations raise the hypothesis that frailty may be associated with distinct cardiac phenotypes in men versus women—a question remains unexplored.

Accordingly, this study aimed to quantify sex-specific associations between physical frailty and CMR-derived cardiac structure, mass, pericardial adiposity, and function in a large population-based cohort. By leveraging CMR’s superior quantitative capabilities, we sought to translate preclinical observations of sex-dimorphic cardiac remodeling to a human population setting, generating hypotheses regarding sex-differentiated cardiovascular phenotypes in frailty.

## Materials and methods

### Study population and study design

The UK Biobank (UKB) (https://www.ukbiobank.ac.uk/) is a large, multicenter, prospective cohort comprising over 500,000 males and females, aged 40–69 years, recruited between 2006 and 2010. All participants provided written informed consent before any data collection. The use of UKB data in the current study is under application number 684,331. The present study evaluates cross-sectional associations using data from the UKB cardiac magnetic resonance (CMR) imaging substudy, with imaging acquired predominantly between 2014 and 2025. Only the first available CMR per participant was included.

**Fig. 1:**
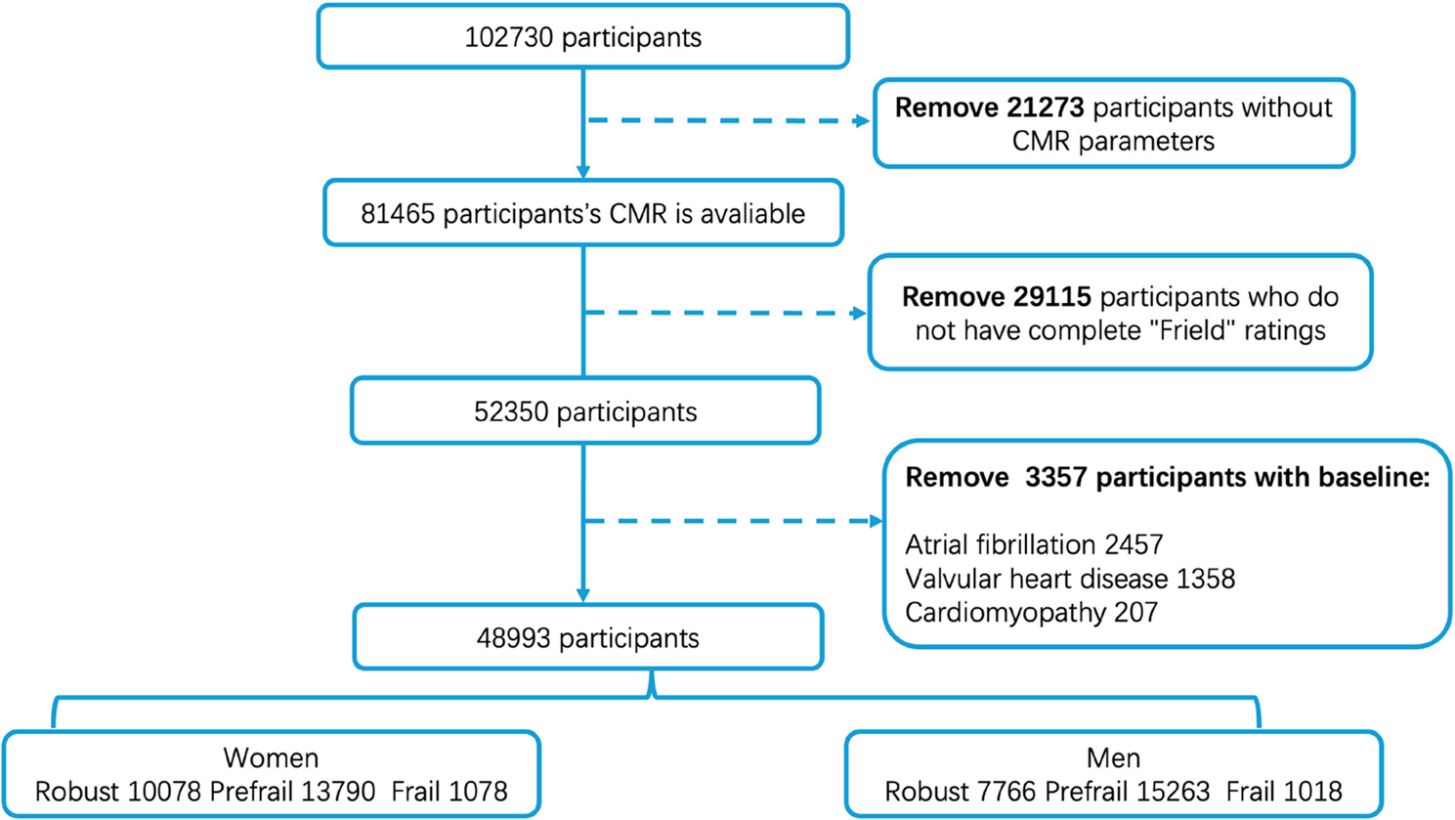
Study population flowchart. Participant inclusion/exclusion and final cohort (N = 48,993). UK Biobank CMR substudy; only the first CMR per participant was included

From 81,465 participants with CMR, eligibility was determined as follows: (1) we excluded participants with missing data in any of the five Fried phenotype components; (2) participants with a documented baseline history of atrial fibrillation, valvular heart disease, or cardiomyopathy were excluded to mitigate potential confounding by pre-existing structural heart disease. The final analytic sample comprised 48,993 participants (Fig. 1). All analyses are cross-sectional and do not imply causality.

### Frailty measurement

Fried originally described and applied the frailty phenotype definition to the Cardiovascular Health Study [16]. Characteristic features include exhaustion, weakness, slowness, physical inactivity, and weight loss (last manifestation). A detailed description of the frailty phenotypes is provided in Table S1. The degree of frailty of the participants is determined by the number of features present: “robust” if none, “pre-frail” if 1–2 features, and “frail” if 3–5 features. Frailty Index (FI) represents another classical approach to frailty, which associated with cardiovascular outcomes [17], However, the Fried phenotype offers distinct advantages, originally developed and validated within the cardiovascular health study [18], it is simpler and more convenient to administer.

### CMR acquisition and quantification

The CMR acquisition and analysis followed UKB protocols [19]. We extracted the following information for analysis: Left Ventricular End-Diastolic Volume (LVEDV), Left Ventricular Mass (LVM), Left Ventricular Wall Thickness (LVWT), Left Ventricular Ejection Fraction (LVEF), LVGLS, Right Ventricular End-Diastolic Volume (RVEDV), Right Ventricular Ejection Fraction (RVEF), Left Atrial Maximum Volume (LAmaxV), Right Atrial Maximum Volume (RAmaxV), Left Atrial Ejection Fraction (LAEF), Right Atrial Ejection Fraction (RAEF). The pericardial fat was derived as described by Bard et al. [20]. Pericardial adipose tissue was automatically quantified using the UKB deep-learning pipeline; this algorithm estimates total pericardial fat and does not distinguish epicardial (intrapericardial) from paracardial (extrapericardial) compartments. Results are therefore reported as total pericardial adiposity. Unless otherwise stated, chamber volumes and LVM were indexed by body surface area (BSA), denoted by the suffix “i”. BSA (m^2) was calculated using the Du Bois and Du Bois formula: BSA was calculated using the Du Bois formula: 0.007184 × height(cm)^0.725 × weight(kg)^0.425. LVGLS was derived from feature tracking on long-axis cine images using the UKB image analysis pipeline; more negative values indicate greater longitudinal shortening. We flagged extreme values lying outside [Q1 − 3×Interquartile Range (IQR), Q3 + 3×IQR] and excluded them from descriptive summaries and regression analyses.

### Covariates

Covariates included age, BMI, smoking status (ever vs. never), Heavy alcohol consumption, waist-to-hip ratio (WHR), and comorbidities (hypertension, heart failure, chronic kidney disease, chronic respiratory disease, diabetes, coronary heart disease, stroke, hyperlipidemia, hypothyroidism, cancer). Disease diagnoses were determined based on self-reported medical histories and International Classification of Diseases, 10th Revision, codes (Table S2). We defined two separate indicators for alcohol consumption as heavy drinker: (1) heavy alcohol consumption was defined as consuming ≥ 30 g/day for men and ≥ 20 g/day for women, and (2) heavy alcohol consumption was also defined based on drinking frequency, with participants who reported drinking ‘Daily or almost daily’ or ‘Three or four times a week’ categorized as heavy drinkers.

### Statistical analysis

The frailty score distribution was highly skewed (Figure S1), with relatively small numbers in the frail category (women *n* = 1078; men *n* = 1018, representing 4.3% and 4.1% of each sex, respectively). Baseline characteristics were compared across all three frailty groups using Kruskal-Wallis tests for continuous variables and chi-square tests for categorical variables, stratified by sex. For regression analyses, frailty was modeled as a three-category variable (robust/pre-frail/frail) with robust as the reference group.

We test the missing data mechanism using Little’s MCAR test and exploratory analysis of missingness predictors. Little’s test yielded χ² = 20,414.63, df = 1,415, *p* < 0.001, rejecting the null hypothesis of missing completely at random (MCAR). Assuming the data were Missing at Random (MAR), we assess the correlation between the missing CMR parameter and baseline characteristics (Figure S2). Then we performed multiple imputation using chained equations to create five imputed datasets. The sensitivity analysis using complete cases yielded effect estimates in the same direction as the primary analysis (Figure S3), providing supportive evidence for the robustness of the multiply imputed results.

We conducted sex-stratified linear regressions for all outcomes, with covariate adjustment (as above), modeling frailty as a three-category variable with robust as the reference category. To quantify sex differences in associations between frailty and the cardiac phenotype, we fit pooled models with sex-frailty interaction terms. Hierarchical models were constructed: Model 1 included sex and frailty main effects only; Model 2 added general health and comorbidities; Model 3 added the sex-frailty interaction (retaining Model 2 covariates). The interaction coefficients represent the difference in effect sizes between men and women (e.g., β_sex-frail = β_male (frail vs. robust) − β_female (frail vs. robust)). Estimates were pooled using Rubin’s rules.

Sensitivity analyses included: (1) complete case analysis to assess robustness to missing data assumptions; (2) delta-adjustment analysis assuming that missing data in women participants had systematically underestimated frailty status (Table S4).

All analyses were conducted using R version 4.3.2 (R Foundation for Statistical Computing), with statistical significance defined as two-sided α < 0.05. Model assumptions were verified using residual diagnostics and variance inflation factors 1.036 ~ 1.341, confirming the absence of multicollinearity.

## Results

### Study population and baseline characteristics

This study included 48,993 participants, comprising 24,946 women and 24,047 men. According to the Fried phenotype classification, 10,078 women (40.4%) were Robust, 13,790 (55.3%) Pre-frail, and 1,078 (4.3%) Frail; among men, 7,766 (31.1%) were Robust, 15,263 (61.1%) Pre-frail, and 1,018 (4.1%) Frail. Compared with the Robust group, the combined Pre-frail and Frail groups exhibited significantly higher body mass index and waist-hip ratio, greater prevalence of ever or current smoking, and heavier burden of chronic diseases (including hypertension, diabetes, heart failure, and chronic kidney disease) in both sexes (all *p* < 0.05). Notably, the prevalence of cancer did not differ significantly between groups (*p* > 0.05) (Table 1).

**Table 1:** Baseline characteristics stratified by sex and frailty status

Characteristic	Women				Men			
	Robust	Pre-frail	Frail	*P*-value	Robust	Pre-frail	Frail	*P*-value
	(*N* = 10,078)	(*N* = 13,790)	(*N* = 1,078)		(*N* = 7,766)	(*N* = 15,263)	(*N* = 1,018)	
Age(y)	64 (59, 70)	63 (57, 69)	64 (58, 70)	< 0.001	66 (60, 71)	65 (59, 71)	68 (61, 73)	< 0.001
BMI(Kg/m2)	24.3 (22.2, 26.8)	25.8 (23.0, 29.2)	29.9 (26.0, 34.0)	< 0.001	25.8 (23.9, 28.1)	26.6 (24.4, 29.2)	28.5 (25.7, 31.9)	< 0.001
WHR	0.81 (0.77, 0.86)	0.83 (0.78, 0.88)	0.86 (0.81, 0.91)	< 0.001	0.93 (0.89, 0.97)	0.94 (0.90, 0.98)	0.98 (0.93, 1.02)	< 0.001
Smoking status				0.015				< 0.001
Never	6,650 (66%)	9,150 (66%)	668 (62%)		4,670 (60%)	9,240 (61%)	528 (52%)	
Ever	3,405 (34%)	4,613 (34%)	407 (38%)		3,082 (40%)	5,992 (39%)	489 (48%)	
Heavy alcohol consumption	4,327 (43%)	5,075 (37%)	283 (26%)	< 0.001	4,249 (55%)	7,948 (52%)	416 (41%)	< 0.001
Hypertension	1,269 (13%)	2,310 (17%)	347 (32%)	< 0.001	1,545 (20%)	3,724 (24%)	429 (42%)	< 0.001
Heart failure	22 (0.2%)	33 (0.2%)	10 (0.9%)	< 0.001	42 (0.5%)	105 (0.7%)	17 (1.7%)	< 0.001
Chronic kidney disease	139 (1.4%)	237 (1.7%)	49 (4.5%)	< 0.001	132 (1.7%)	329 (2.2%)	46 (4.5%)	< 0.001
Chronic respiratory disease	776 (7.7%)	1,348 (9.8%)	198 (18%)	< 0.001	583 (7.5%)	1,249 (8.2%)	164 (16%)	< 0.001
Diabetes mellitus	145 (1.4%)	458 (3.3%)	118 (11%)	< 0.001	252 (3.2%)	903 (5.9%)	163 (16%)	< 0.001
Coronary Heart Disease	256 (2.5%)	453 (3.3%)	86 (8.0%)	< 0.001	556 (7.2%)	1,308 (8.6%)	133 (13%)	< 0.001
Stroke	123 (1.2%)	192 (1.4%)	31 (2.9%)	< 0.001	165 (2.1%)	326 (2.1%)	42 (4.1%)	< 0.001
Hyperlipidemia	549 (5.4%)	1,004 (7.3%)	141 (13%)	< 0.001	890 (11%)	2,000 (13%)	217 (21%)	< 0.001
Hypothyroidism	576 (5.7%)	969 (7.0%)	121 (11%)	< 0.001	129 (1.7%)	326 (2.1%)	34 (3.3%)	< 0.001
Cancer	130 (1.3%)	204 (1.5%)	20 (1.9%)	0.2	96 (1.2%)	195 (1.3%)	17 (1.7%)	0.5
LVWT(mm)	5.14 (4.83, 5.48)	5.19 (4.87, 5.56)	5.33 (4.97, 5.76)	< 0.001	6.12 (5.74, 6.52)	6.12 (5.73, 6.56)	6.21 (5.76, 6.71)	< 0.001
LVMi(g/m²)	40.3 (37.0, 44.0)	39.9 (36.5, 43.6)	39.2 (36.0, 43.4)	< 0.001	51 (46, 55)	50 (45, 55)	49 (44, 54)	< 0.001
LVEDVi(ml/m²)	74 (67, 82)	73 (66, 80)	70 (63, 78)	< 0.001	84 (76, 94)	82 (73, 91)	78 (69, 87)	< 0.001
LVEF(%)	61.5 (57.8, 65.1)	61.4 (57.8, 65.0)	61.3 (57.8, 65.4)	0.7	58.1 (54.3, 61.9)	58.3 (54.6, 62.2)	58.8 (54.8, 63.1)	< 0.001
LVGLS(%)	-18.99 (-20.61, -17.41)	-19.12 (-20.83, -17.51)	-19.24 (-20.62, -17.66)	< 0.001	-17.87 (-19.47, -16.35)	-17.91 (-19.57, -16.33)	-17.91 (-19.54, -16.28)	0.7
LAmaxVi(ml/m²)	38 (32, 45)	38 (32, 44)	38 (31, 44)	0.001	38 (31, 46)	37 (30, 45)	37 (29, 44)	< 0.001
LAEF(%)	62 (57, 67)	61 (57, 66)	60 (56, 65)	< 0.001	61 (56, 66)	61 (56, 66)	60 (54, 65)	0.002
RAmaxVi(ml/m²)	44 (38, 52)	42 (35, 49)	38 (32, 46)	< 0.001	48 (40, 58)	46 (38, 55)	42 (34, 51)	< 0.001
RAEF(%)	49 (43, 54)	49 (44, 54)	49 (44, 56)	< 0.001	44 (39, 49)	44 (39, 50)	46 (40, 51)	< 0.001
RVEDVi(ml/m²)	78 (70, 86)	76 (69, 84)	73 (66, 81)	< 0.001	92 (82, 102)	89 (79, 99)	84 (74, 93)	< 0.001
RVEF(%)	59.3 (55.7, 62.8)	59.4 (55.9, 62.9)	59.3 (55.9, 62.6)	0.15	55.7 (52.1, 59.3)	55.6 (51.9, 59.1)	55.9 (52.2, 59.6)	0.051
Pericardial Fat(cm^2^)	16 (11, 21)	17 (12, 24)	20 (14, 27)	< 0.001	24 (17, 33)	26 (18, 35)	30 (21, 39)	< 0.001

Values are median (IQR), or n (%). Female/male refer to biological sex at assessment

*Abbreviations*: *BMI* Body Mass Index, *WHR* Waist-to-Hip Ratio, *LVWT* Left Ventricular Wall Thickness, *LVMi* Left Ventricular Mass Index, *LVEDVi* Left Ventricular End-Diastolic Volume Index, *LVEF* Left Ventricular Ejection Fraction, *LVGLS* Left Ventricular Global Longitudinal Strain, *LAmaxVi* Left Atrial Maximum Volume Index, *LAEF* Left Atrial Ejection Fraction, *RAmaxVi* Right Atrial Maximum Volume Index, *RAEF* Right Atrial Ejection Fraction, *RVEDVi* Right Ventricular End-Diastolic Volume Index, *RVEF* Right Ventricular Ejection Fraction

*P* values from Kruskal-Wallis tests or χ^2 test, as appropriate.

### Cardiac structure

**Fig. 2:**
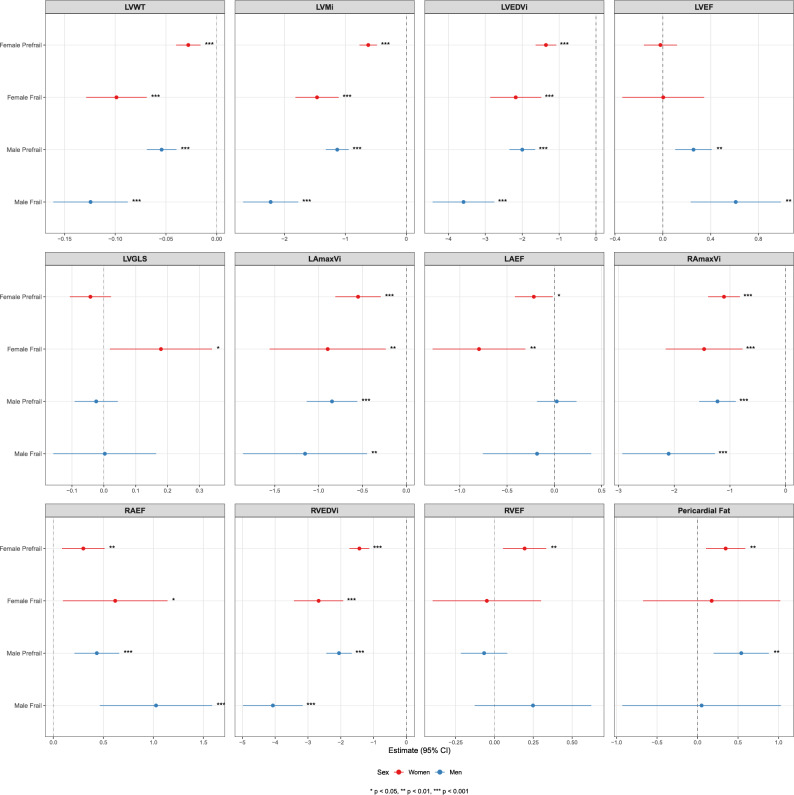
Sex-stratified associations between frailty score and cardiac parameters

Sex-stratified analyses revealed consistent negative associations between frailty and cardiac chamber volumes, left ventricular mass, and wall thickness in both sexes (Table 2; Fig. 2).

**Table 2:** Sex-stratified associations between frailty score and cardiac parameters

	Women				Men			
	Pre-frail vs. Robust		Frail vs. Robust		Pre-frail vs. Robust		Frail vs. Robust	
Parameter	β ± SE	*P* value	β ± SE	*P* value	β ± SE	*P* value	β ± SE	*P* value
LVWT (mm)	-0.028 (0.006)	< 0.001	-0.099 (0.015)	< 0.001	-0.054 (0.007)	< 0.001	-0.124 (0.019)	< 0.001
LVMi (g/m²)	-0.625 (0.073)	< 0.001	-1.465 (0.181)	< 0.001	-1.134 (0.095)	< 0.001	-2.227 (0.231)	< 0.001
LVEDVi (ml/m²)	-1.357 (0.142)	< 0.001	-2.176 (0.352)	< 0.001	-1.999 (0.178)	< 0.001	-3.591 (0.427)	< 0.001
LVEF (%)	-0.020 (0.071)	0.779	0.004 (0.175)	0.983	0.257 (0.078)	0.001	0.610 (0.193)	0.002
LVGLS (%)	-0.042 (0.033)	0.202	0.179 (0.082)	0.028	-0.024 (0.034)	0.488	0.003 (0.082)	0.971
LAmaxVi (ml/m²)	-0.550 (0.132)	< 0.001	-0.896 (0.337)	0.008	-0.847 (0.147)	< 0.001	-1.154 (0.361)	0.001
LAEF (%)	-0.217 (0.102)	0.034	-0.797 (0.250)	0.002	0.025 (0.106)	0.813	-0.183 (0.292)	0.533
RAmaxVi (ml/m²)	-1.106 (0.145)	< 0.001	-1.463 (0.352)	< 0.001	-1.223 (0.167)	< 0.001	-2.100 (0.424)	< 0.001
RAEF (%)	0.299 (0.108)	0.006	0.618 (0.267)	0.021	0.434 (0.114)	< 0.001	1.026 (0.286)	< 0.001
RVEDVi (ml/m²)	-1.431 (0.154)	< 0.001	-2.674 (0.383)	< 0.001	-2.054 (0.197)	< 0.001	-4.069 (0.464)	< 0.001
RVEF (%)	0.194 (0.071)	0.006	-0.049 (0.178)	0.782	-0.067 (0.076)	0.379	0.248 (0.191)	0.196
Pericardial Fat (cm^2^)	0.347 (0.124)	0.008	0.175 (0.432)	0.695	0.540 (0.174)	0.004	0.050 (0.499)	0.921

Adjusted for age, BMI, smoking (ever vs. never), heavy alcohol consumption, WHR, hypertension, heart failure, chronic kidney disease, chronic respiratory disease, diabetes, coronary heart disease, stroke, hyperlipidemia, hypothyroidism, and cancer. Analysis based on multiply imputed data (*N* = 48,993)

*LVWT* Left Ventricular Wall Thickness, *LVMi* Left Ventricular Mass Index, *LVEDVi* Left Ventricular End-Diastolic Volume Index, *LVEF* Left Ventricular Ejection Fraction, *LVGLS* Left Ventricular Global Longitudinal Strain, *LAmaxVi* Left Atrial Maximum Volume Index, *LAEF* Left Atrial Ejection Fraction, *RAmaxVi* Right Atrial Maximum Volume Index, *RAEF* Right Atrial Ejection Fraction, *RVEDVi* Right Ventricular End-Diastolic Volume Index, *RVEF* Right Ventricular Ejection Fraction

#### Ventricular mass and volumes

In women, pre-frailty was associated with lower LVMi (β = − 0.625 g/m², *p* < 0.001), smaller LVEDVi (β = − 1.357 mL/m², *p* < 0.001), and smaller RVEDVi (β = − 1.431 mL/m², *p* < 0.001) compared with robust individuals. These differences were more pronounced in the frail stage (LVMi: β = − 1.465 g/m²; LVEDVi: β = − 2.176 mL/m²; RVEDVi: β = − 2.674 mL/m²; all *p* < 0.001). LVWT was also smaller in women with frailty (pre-frail: β = − 0.028 mm, p 0.001; frail: β = − 0.099 mm, *p* < 0.001).

In men, the associations were considerably stronger. Compared with robust men, those with pre-frailty had lower LVMi (β = − 1.134 g/m²), smaller LVEDVi (β = − 1.999 mL/m²), and smaller RVEDVi (β = − 2.054 mL/m²) (all *p* < 0.001). These differences were even larger in frail men (LVMi: β = − 2.227 g/m²; LVEDVi: β = − 3.591 mL/m²; RVEDVi: β = − 4.069 mL/m²; all *p* < 0.001). LVWT was also smaller in pre-frail (β = − 0.054 mm, *p* < 0.001) and frail men (β = − 0.124 mm, *p* < 0.001).

Sex-by-frailty interaction analyses (Table 3, Model 3) confirmed that the structural differences associated with frailty were significantly more pronounced in men across multiple indices. The interaction term for LVEDVi was β = − 1.134 (*p* < 0.001) at the pre-frail stage and β = − 1.455 (*p* < 0.001) at the frail stage, indicating that the difference between frail and robust individuals was 1.134–1.455 mL/m² larger in men than in women. Similarly, significant interactions were observed for LVMi (pre-frail: β = − 0.449, *p* < 0.001; frail: β = − 0.895, *p* = 0.002) and RVEDVi (pre-frail: β = − 1.133, *p* < 0.001; frail: β = − 3.323, *p* < 0.001), demonstrating a consistent pattern of stronger associations in men. In contrast, the interaction for LVWT did not reach statistical significance (*p* > 0.05), suggesting that the slightly larger absolute difference in men reflects proportional differences similar to those in women after accounting for baseline sex differences.

**Table 3:** Multivariable associations of sex and frailty with cardiac structure/function

Parameter	Model	*R* ^2^	Pre-frail vs. Robust β (SE)	Frail vs. Robust β (SE)	Sex×Pre-frail *p*	Sex×Frail *p*
LVWT(mm)	Model 1	0.414	0.036 (0.005)	0.160 (0.013)	-	-
	Model 2	0.54	-0.045 (0.005)	-0.122 (0.012)	-	-
	Model 3	0.54	-0.044 (0.007)	-0.129 (0.016)	0.786	0.554
LVMi(g/m²)	Model 1	0.394	-0.699 (0.060)	-1.361 (0.144)	-	-
	Model 2	0.415	-0.892 (0.060)	-1.900 (0.146)	-	-
	Model 3	0.415	-0.681 (0.082)	-1.474 (0.201)	< 0.001	0.002
LVEDVi(ml/m²)	Model 1	0.13	-2.240 (0.117)	-5.710 (0.284)	-	-
	Model 2	0.209	-1.619 (0.113)	-2.773 (0.277)	-	-
	Model 3	0.21	-1.134 (0.154)	-1.455 (0.379)	< 0.001	< 0.001
LVEF(%)	Model 1	0.076	0.102 (0.052)	0.386 (0.127)		-
	Model 2	0.085	0.107 (0.052)	0.285 (0.131)	-	-
	Model 3	0.085	-0.028 (0.072)	-0.007 (0.177)	0.006	0.015
LVGLS(%)	Model 1	0.052	-0.090 (0.024)	-0.087 (0.057)	-	-
	Model 2	0.062	-0.042 (0.024)	0.073 (0.058)	-	-
	Model 3	0.062	-0.082 (0.033)	0.060 (0.080)	0.076	0.797
LAmaxVi(ml/m²)	Model 1	0.001	-0.638 (0.098)	-1.049 (0.243)	-	-
	Model 2	0.019	-0.692 (0.098)	-1.036 (0.246)	-	-
	Model 3	0.019	-0.567 (0.134)	-0.949 (0.339)	0.174	0.693
LAEF(%)	Model 1	0.003	-0.189 (0.073)	-1.201 (0.184)	-	-
	Model 2	0.025	-0.081 (0.073)	-0.447 (0.185)	-	-
	Model 3	0.025	-0.114 (0.102)	-0.486 (0.249)	0.636	0.83
RAmaxVi(ml/m²)	Model 1	0.038	-2.439 (0.114)	-6.173 (0.278)	-	-
	Model 2	0.147	-1.145 (0.108)	-1.733 (0.270)	-	-
	Model 3	0.148	-0.978 (0.149)	-0.986 (0.365)	0.1	0.004
RAEF(%)	Model 1	0.072	0.508 (0.076)	1.348 (0.190)		
	Model 2	0.079	0.370 (0.077)	0.843 (0.194)		
	Model 3	0.079	0.294 (0.107)	0.520 (0.262)	0.305	0.08
RVEDVi(ml/m²)	Model 1	0.199	-2.390 (0.128)	-6.726 (0.305)		
	Model 2	0.273	-1.682 (0.124)	-3.254 (0.299)		
	Model 3	0.274	-1.153 (0.167)	-1.661 (0.412)	< 0.001	< 0.001
RVEF(%)	Model 1	0.107	0.008 (0.051)	0.062 (0.126)		
	Model 2	0.116	0.074 (0.052)	0.109 (0.129)		
	Model 3	0.117	0.206 (0.070)	-0.013 (0.175)	0.007	0.355
Pericardial Fat(cm^2^)	Model 1	0.169	1.790 (0.125)	5.047 (0.377)		
	Model 2	0.349	0.419 (0.118)	0.085 (0.391)		
	Model 3	0.349	0.202 (0.138)	-0.358 (0.456)	0.012	0.079

Model 1: sex and the degree of frailty only. Model 2: Model 1 + general health and comorbidities. Model 3: Model 2 + sex × degree of frailty. Model 3 coefficients represent the additional effect in males compared with females (male effect - female effect. Analysis based on multiply imputed data (*N* = 48,993)

*LVWT* Left Ventricular Wall Thickness, *LVMi* Left Ventricular Mass Index, *LVEDVi* Left Ventricular End-Diastolic Volume Index, *LVEF* Left Ventricular Ejection Fraction, *LVGLS* Left Ventricular Global Longitudinal Strain, *LAmaxVi* Left Atrial Maximum Volume Index, *LAEF* Left Atrial Ejection Fraction, *RAmaxVi* Right Atrial Maximum Volume Index, *RAEF* Right Atrial Ejection Fraction, *RVEDVi* Right Ventricular End-Diastolic Volume Index, *RVEF* Right Ventricular Ejection Fraction

#### Atrial volumes

Similar to ventricular findings, frailty was associated with smaller atrial volumes in both sexes (women: pre-frail and frail; men: pre-frail and frail; all *p* < 0.05). For RAmaxVi, a significant interaction emerged only at the frail stage (β = − 0.986, *p* = 0.004), suggesting that sex differences in right atrial associations become evident only with advanced frailty. However, unlike ventricular indices, no significant sex-by-frailty interactions were observed for left atrial volumes.

### Cardiac function

Functional parameters exhibited markedly distinct sex-specific patterns, with men showing a positive association and women demonstrating negative associations with frailty.

#### LVEF

LVEF showed no significant association with frailty in women (pre-frail: β = − 0.020, *p* = 0.779; frail: β = 0.004, *p* = 0.983). In men, however, a higher frailty stage was associated with progressively higher LVEF (pre-frail: β = 0.257, *p* = 0.001; frail: β = 0.610, *p* = 0.002). The sex-by-frailty interaction was significant at both stages (pre-frail: β =-0.028, *p* = 0.006; frail: β =-0.007, *p* = 0.015), confirming that the positive LVEF–frailty association is specific to men. This finding may reflect a compensatory response to maintain cardiac output in the setting of smaller ventricular volumes.

#### LVGLS

LVGLS was similar across frailty stages in men but was less negative (indicating impaired function) in frail women (β = 0.179, *p* = 0.028). Sensitivity analyses, accounting for the low missing rate of LVGLS (4.7%), confirmed that the association in women was present only at the frail stage and remained robust under moderate missing-data assumptions (delta 0.1–0.2, both *p* < 0.05), with attenuation only under the most extreme assumption (delta 0.3, *p* = 0.140) (Table S4).

#### Left and right atrial function

LAEF was progressively lower with higher frailty stage in women (pre-frail: β = − 0.217, *p* = 0.034; frail: β = − 0.797, *p* = 0.002), whereas no association was observed in men (pre-frail: β = 0.025, *p* = 0.813; frail: β = − 0.183, *p* = 0.533). The interaction terms were not significant (*p* > 0.6), indicating that the apparent sex difference reflects a strong within-female effect rather than a true cross-sex differential. In contrast to left atrial function, RAEF was positively associated with frailty in both sexes (women, pre-frail: β = 0.299, *p* = 0.006; frail: β = 0.618, *p* = 0.021; men, pre-frail: β = 0.434, *p* < 0.001; frail: β = 1.026, *p* < 0.001), with no significant sex interaction.

#### RVEF

RVEF was positively associated with frailty only in pre-frail women (β = 0.194, *p* = 0.006), with a significant sex interaction at the pre-frail stage (β = 0.206, *p* = 0.007). However, this association and the corresponding interaction were absent in the frail stage, suggesting that the sex difference in RVEF is confined to early frailty.

### Pericardial fat

The association between pericardial fat volume and frailty exhibited a distinct stage-dependent pattern. In both sexes, pericardial fat was higher in the pre-frail stage compared with robust individuals (women: β = 0.347, *p* = 0.008; men: β = 0.540, *p* = 0.004). However, this association was no longer observed in the frail stage (women: β = 0.175, *p* = 0.695; men: β = 0.050, *p* = 0.921). indicating a stage-dependent relationship between pericardial fat and frailty severity. Although pericardial fat had a relatively high missing rate (33.2%), sensitivity analyses using delta adjustment (0.1–0.3) confirmed the robustness of this pattern: the positive association in pre-frail women remained significant across all scenarios, while the null association in frail women remained consistently non-significant.

Sex-by-frailty interaction analysis revealed a significant interaction only at the pre-frail stage (β = 0.462, *p* = 0.012), indicating that the difference in pericardial fat between pre-frail and robust individuals was greater in men than in women. The interaction at the frail stage was marginal (β = 0.930, *p* = 0.079).

### Sensitivity analyses

Multiple sensitivity analyses confirmed the robustness of these findings. Complete-case analysis reproduced the main estimates for LVEDVi, LVMi, RVEDVi, LVEF, and other key indices (Supplement Fig. 3), and delta-adjustment analyses confirmed the robustness of findings for LVGLS, LAEF, and pericardial fat in women (Supplement Table 4), indicating that missing data did not materially bias the results.

## Discussion

Our findings reveal distinct sex-specific patterns in the association between physical frailty and CMR-derived cardiac phenotypes. In men, progression from robust to pre-frail to frail was associated with progressively smaller cardiac chamber volumes and lower left ventricular mass in a graded, dose-dependent manner (e.g., LVMi: pre-frail β = − 1.134, frail β = − 2.227; LVEDVi: pre-frail β = − 1.999, frail β = − 3.591; all *p* < 0.001). Notably, LVEF was progressively higher with greater frailty severity (pre-frail β = 0.257, frail β = 0.610, both *p* < 0.01), whereas LVGLS was similar to robust individuals even in frail men. In women, frailty was similarly associated with smaller chamber volumes and lower LV mass. However, significant sex-by-frailty interactions were observed for LVMi, LVEDVi, and RVEDVi (all *p* < 0.001), indicating more pronounced ventricular structural differences in men. The distinguishing feature in women was poorer functional parameters: LAEF was lower in both pre-frail (β = − 0.217, *p* = 0.034) and frail stages (β = − 0.797, *p* = 0.002), and LVGLS was less negative (indicating impaired strain) specifically in frail women (β = 0.179, *p* = 0.028). Pericardial fat was higher in the pre-frail stage (men: β = 0.540, *p* = 0.004; women: β = 0.347, *p* = 0.008) but was not significantly different between frailty and robust individuals in either sex.

Our study found that physical frailty was associated with smaller LVMi and LVEDVi in both men and women, with consistent trends observed in both pre-frail and frail stages. However, significant sex × frailty interactions were observed for these parameters (*p* < 0.05), suggesting that men are more susceptible to frailty-related left ventricular structural remodeling. Although we pay more attention to the increase in the cardiac chamber volume in clinical practice, we ignore the clinical results caused by its reduction. As a key feature of frailty, reduced physical activity may decrease plasma volume and preload [21], possibly via altered neurohormonal tone, which in turn leads to smaller end-diastolic volumes. Consistent with this, Thomas et al. [22] showed that higher device-measured physical activity is associated with higher LVEDVi in UKB (women: +0.70 mL/m², men: +1.08 mL/m²). These findings imply that the male heart may be more sensitive to declines in physical activity, possibly due to higher basal metabolic demands. Although this study does not directly validate our findings, it provides indirect support for sex differences in frailty-related cardiac remodeling. Although aging is not synonymous with frailty, the two are closely interrelated. In a clinical CMR study also based on the UK Biobank [23], accelerated biological aging was associated with reduced cardiac chamber volumes in both sexes. However, the risk of heart failure associated with accelerated aging was more pronounced in women. Nevertheless, animal studies have provided insights into the sex-specific biology of the aging heart that may inform our understanding of frailty. Notably, Zhang’s study [24] demonstrated that male hearts are characterized by cardiomyocyte loss and reduced cardiac mass—changes that insufficient compensatory hypertrophy fails to offset. In contrast, females preserve cardiac mass through enhanced cell survival and low apoptosis, despite diminished proliferative capacity. This suggests that fundamental sex differences in cardiac aging may underlie the distinct patterns of frailty-associated remodeling observed in our study.

Previous studies have paid limited attention to the structural and functional abnormalities of the right heart caused by frailty. We observed smaller right-sided volumes (RVEDVi, RAmaxVi) and higher RAEF with greater frailty, in both sexes. Because right atrial pump function is less preload-dependent than LV contractility [25], a higher RAEF could represent compensation to maintain forward flow under lower preload. However, measurement variability and loading conditions are alternative explanations. Longitudinal imaging with loading assessments is needed to clarify mechanisms.

We postulate that the increase in LVEF observed exclusively in frail men represents a compensatory response to maintain cardiac output in the setting of reduced preload (LVEDVi). The decline in physical activity accompanying frailty may reduce LVEDVi; in men, this appears to enhance systolic performance through increased ejection fraction, possibly reflecting the mobilization of cardiac reserve. In contrast, women did not exhibit this response, which may be attributable to their relatively smaller reduction in LVEDVi compared to men. This less pronounced decline in preload may obviate the need for a compensatory increase in LVEF. Alternatively, women may adopt distinct hemodynamic strategies—such as heart rate modulation—to adapt to frailty-related preload reduction, rather than relying on enhanced contractility. Another possibility is survival selection, where males with lower LVEF may have dropped out or experienced higher mortality during the transition from pre-frailty to frailty, leaving relatively healthier survivors.

In women, higher frailty was associated with lower LAEF and a less-negative LVGLS. A lower absolute LVGLS primarily reflects impaired longitudinal systolic deformation; it has also been associated with abnormalities in diastolic function [26]. In the context of reduced LAEF with largely preserved chamber volumes and LVEF, this pattern is compatible with—but not diagnostic of—early HFpEF-related physiology. However, we lacked direct diastolic measures (e.g., filling pressures), so mechanistic inferences remain speculative.LA dysfunction is closely linked to HFpEF and may precede overt symptoms [27, 28]. This finding may partly explain why older women are more likely to develop HFpEF [29]. Sex differences exist in the impact of genetic and environmental factors on cardiac structure-function relationships, such as hormones, inflammatory status, and endothelial function [30]. Females tend to exhibit higher diffuse interstitial fibrosis (as reflected by elevated extracellular volume fraction), whereas males more frequently show focal replacement fibrosis (nonischemic late gadolinium enhancement) [31]. The predisposition of women to diffuse interstitial fibrosis may underlie their vulnerability to myocardial strain [32]. This provides a reasonable explanation for functional changes preceding structural remodeling in women with physical frailty.

In our study, pericardial fat area was positively associated with frailty degree in pre-frail individuals of both sexes; however, a significant sex interaction was observed. After adjusting for age and BMI, men exhibited a more pronounced increase in pericardial fat during the pre-frail stage compared to women. Notably, this independent association and the sex-specific interaction were no longer evident in the frail group. Although our baseline data indicated that men had larger pericardial fat areas than women—consistent with findings by Joel T. Rämö et al. [33]—and showed an initial rise in fat area with increasing frailty severity, this trend disappeared after adjusting for BMI, age, lifestyle factors, and comorbidities in the frail population. This nonlinear trajectory suggests that pericardial fat accumulation may plateau at advanced stages of frailty, with the association particularly pronounced in men. Prior research in patients with depression has reported a similar sex-specific pattern, where depression correlated with increased pericardial fat accumulation in men but not in women [34], suggesting that male pericardial fat may be more sensitive to psychosocial stressors. Given that depression strongly correlates with core features of the frailty phenotype (e.g., low physical activity, fatigue, and slow gait speed) [35]. That reduced physical activity appears to mediate the link between depression and pericardial fat [36]. These mechanisms may partly explain our findings. Furthermore, pericardial fat volume is known to correlate with coronary plaque burden, with stronger associations reported in men [37]. This raises the possibility that greater pericardial fat accumulation confers a higher risk of atherosclerotic plaque burden, specifically in men.

Supporting this hypothesis, our data revealed a higher prevalence of coronary heart disease (CHD) in the frail group compared to the robust group across both sexes, with frail men exhibiting the highest prevalence among all subgroups (Pre-frail: 8.6% vs. Frail: 13%). Although our study is cross-sectional, these findings align with a male-predominant vulnerability linking frailty, pericardial fat, and coronary disease. Given that epicardial/pericardial fat is modifiable, lifestyle interventions—such as regular physical activity and adherence to a Mediterranean diet—represent plausible targets for risk mitigation, as they have been associated with reductions in these fat depots [38, 39]. While these strategies show promise, prospective studies are needed to confirm causality and validate their efficacy.

This study has several limitations that should be considered when interpreting the findings. First, the cross-sectional design precludes establishing causal relationships or temporal sequence, and reverse causation cannot be excluded. Second, selection bias and survivor bias are important concerns: UK Biobank participants are generally healthier than the broader population, and the prevalence of frailty in our sample (4.1–4.3%) was substantially lower than the 10–15% reported in community-dwelling older adults. Critically, individuals with severe frailty may have been unable to tolerate CMR scanning or may have already died from cardiovascular events before imaging assessment, resulting in a sample skewed toward relatively healthy survivors. This bias likely underestimates the true strength of associations between frailty and cardiac abnormalities, particularly masking extreme phenotypes in the most frail individuals. Third, pericardial fat quantification did not distinguish epicardial from paracardial adipose tissue, and the relative contributions of these compartments may differ by sex, potentially diluting biological associations. Fourth, the absence of tissue characterization parameters (T1 mapping, extracellular volume fraction) and direct diastolic measures (e.g., filling pressures) limits mechanistic insights, particularly regarding our hypothesis that functional changes precede structural remodeling in women.

## Conclusions

This study demonstrates that physical frailty is associated with distinct, sex-specific cardiac phenotypes as assessed by CMR. In men, greater frailty severity was associated with a graded, dose-dependent pattern of smaller cardiac chamber volumes and lower left ventricular mass, accompanied by higher LVEF—a pattern suggestive of a compensatory response to reduced preload. In women, frailty was similarly associated with smaller chamber volumes and lower LV mass, but these associations were significantly weaker than in men. The distinguishing feature in women was poorer functional parameters: lower LAEF and impaired LVGLS specifically in frail women, a pattern compatible with early HFpEF-related physiology. These findings highlight that frailty-associated cardiac remodeling is not a uniform process; men exhibit more pronounced structural differences, while women demonstrate greater functional impairment. Pericardial fat followed a non-linear, stage-dependent pattern: it was higher in the pre-frail stage in both sexes, with a more pronounced increase in men, but these differences were not present in the frail stage. The higher CHD prevalence observed in frail men aligns with a male-predominant vulnerability linking frailty, pericardial fat, and coronary disease.

These findings provide novel insights into the sex-specific cardiac manifestations of physical frailty and may help explain why older men and women are susceptible to different cardiovascular diseases. The observed patterns suggest that early intervention—particularly in the pre-frail stage—may offer a window of opportunity to mitigate risk.

## Supplementary Information

Supplementary Material 1.

## Data Availability

The data that support the findings of this study are available from the UK Biobank resource (www.ukbiobank.ac.uk), but they are not publicly available. Researchers can apply to access the data by registering on the UK Biobank website.

## Abbreviations

BMI: Body mass index
BSA: Body surface area
CHD: Coronary heart disease
CMR: Cardiac magnetic resonance
FI: Frailty index
HFpEF: Heart failure with preserved ejection fraction
IQR: Interquartile range
LAEF: Left atrial ejection fraction
LAmaxV: Left atrial maximum volume
LVEF: Left ventricular ejection fraction
LVEDV: Left ventricular end-diastolic volume
LVGLS: Left ventricular global longitudinal strain
LVM: Left ventricular mass
LVWT: Left ventricular wall thickness
MAR: Missing at random
MCAR: Missing completely at random
MBP: Mean blood pressure
RAmaxV: Right atrial maximum volume
RAEF: Right atrial ejection fraction
RVEF: Right ventricular ejection fraction
RVEDV: Right ventricular end-diastolic volume
SE: Standard error
UKB: United kingdom biobank
WHR: Waist-to-hip ratio
