# Supplementary material for: Sex-specific associations of physical frailty with cardiac structure and function: a cross-sectional study based on UK Biobank
Source: BMC Cardiovasc Disord. 2026 Apr 9;26:496. doi: 10.1186/s12872-026-05737-5 (PMC13255337; doi:10.1186/s12872-026-05737-5)
Supplement: Supplementary file 1 — Supplementary Material 1. [file 12872_2026_5737_MOESM1_ESM.docx]

**Supplemental Material**

**Supplement TABLE 1 Fried** **phenotype and corresponding fields in the UK Biobank**

| **Fried** | **UKB Data-Field** | **Self-reported question/measurement in UK Biobank** | **Specific standards** |
| --- | --- | --- | --- |
| Exhaustion | 2080 | Frequency of tiredness / lethargy in last 2 weeks | More than half the days/Nearly every day |
| Weakness | 46 or 47 | Hand grip strength left or right  (Select the larger values) | Male:BMI <= 24.0 & strength <= 29.0,  24.0< BMI <= 28.0 & strength <= 30.0,  BMI > 28.0 & strength <= 32.0  Female: BMI <= 23.0 & strength <= 17.0,  23.0 <BMI <= 26.0 & strength <= 17.3,  26.0<BMI<= 29.0 & strength <= 18.0,  BMI > 29.0 & strength <= 21.0 |
| Slowness | 924 | Usual walking pace | Slow pace~ 1 |
| Physical inactivity | 22040 | Summed MET minutes per week for all activity | Male < 600 ~ 1  Female < 450 ~ 1 |
| Weight loss | 2306 | Weight change compared with 1 year ago | Yes - lost weight |

BMI：Body mass index；MET：metabolic equivalent of task

**Supplement TABLE 2 Disease corresponding ICD-10 codes and Self-report fields**

| **Diseses** | **ICD-10** | **Self-report** |
| --- | --- | --- |
| atrial fibrillation | I48 | (?i)atrial fibrillation\|atrial fib\|af |
| valvular heart disease | I05 I06 I07 I08 I09 I34 I35 I36 I37 I38 | (?i)valve |
| cardiomyopathy | I42 I43 | (?i)cardiomyopathy |
| hypertension | I10 I11 I12 I13 I15 I12.0 I13.1 | (?i)hypertension |
| coronary heart disease | I20 I21 I22 I23 I24 I25 | (?i)angina\|heart attack\|myocardial infarction |
| heart failure | I50 I11.0 I13.0 I13.2 | (?i)heart failure\|cardiac failure |
| diabetes mellitus | E10 E11 E12 E13 E14 | (?i)diabetes |
| renal failure | N18 N19 | (?i)kidney failure\|renal failure |
| chronic respiratory disease | J40 J41 J42 J43 J44 J45 J46 J47 J60 J61 J62 J63 J64 J65 J66 J67 J68 J69 J70 J80 J81 J82 J83 J84 J96.1 | (?i)copd\|asthma\|emphysema\|chronic bronchitis |
| stroke | I60 I61 I62 I63 I64 I65 I66 I67 I68 I69 | (?i)stroke\|cerebrovascular\|brain attack |
| hyperlipidemia | E78.0 E78.1 E78.2 E78.3 E78.4 E78.5 | (?i)hyperlipidemia\|high cholesterol |
| hypothyroidism | E03 E89.0 | (?i)hypothyroidism\|underactive thyroid |
| Cancer | C0~26 C30~97 D37~48 | cancer\|tumor\|malign\|onco\|neoplasm\|carcinoma |

**Supplement Table 3 Percentage of Missing Values**

| **Variables** | **Missing count (total number=48,993)** | **Missing rate (%)** |
| --- | --- | --- |
| WHR | 6 | 0.01 |
| Smoking status | 99 | 0.2 |
| Drink_freq | 7 | 0.01 |
| LVWT | 780 | 1.59 |
| LVMi | 738 | 1.51 |
| LVEDVi | 998 | 2.04 |
| LVEF | 916 | 1.87 |
| LVGLS | 2301 | 4.7 |
| LAmaxVi | 3005 | 6.13 |
| LAEF | 3563 | 7.27 |
| RAmaxVi | 3336 | 6.81 |
| RAEF | 2937 | 5.99 |
| RVEDVi | 809 | 1.65 |
| RVEF | 924 | 1.89 |
| Pericardial Fat | 16284 | 33.24 |

**Supplement Figure** **1 Distribution of Frailty Scores**

**
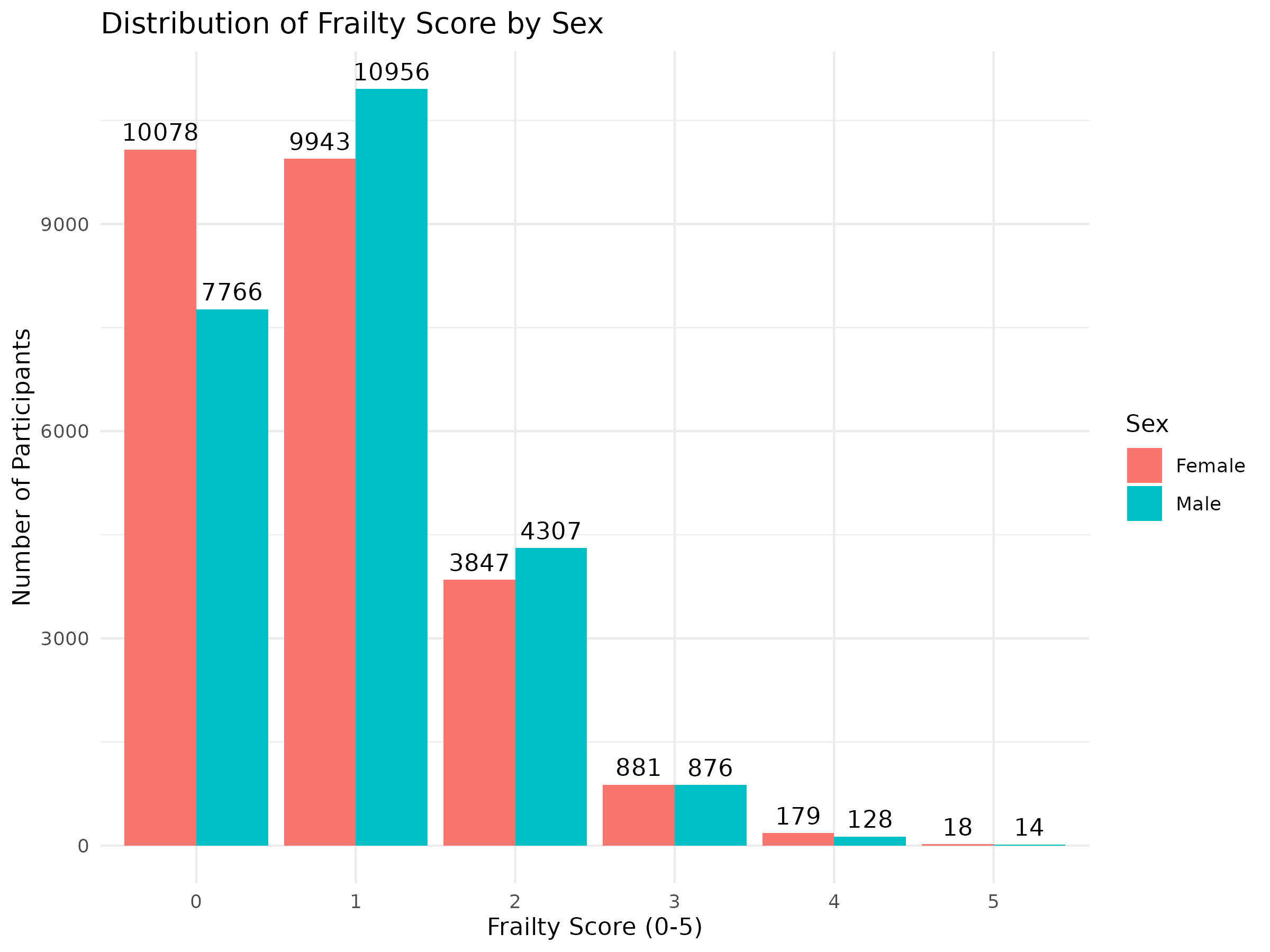
**

Bar chart illustrating the frequency distribution of frailty scores across the study population. The distribution provides insight into the prevalence and severity of frailty in the cohort.

**Supplement Figure 2 Missing Values Correlation Heatmap**

**
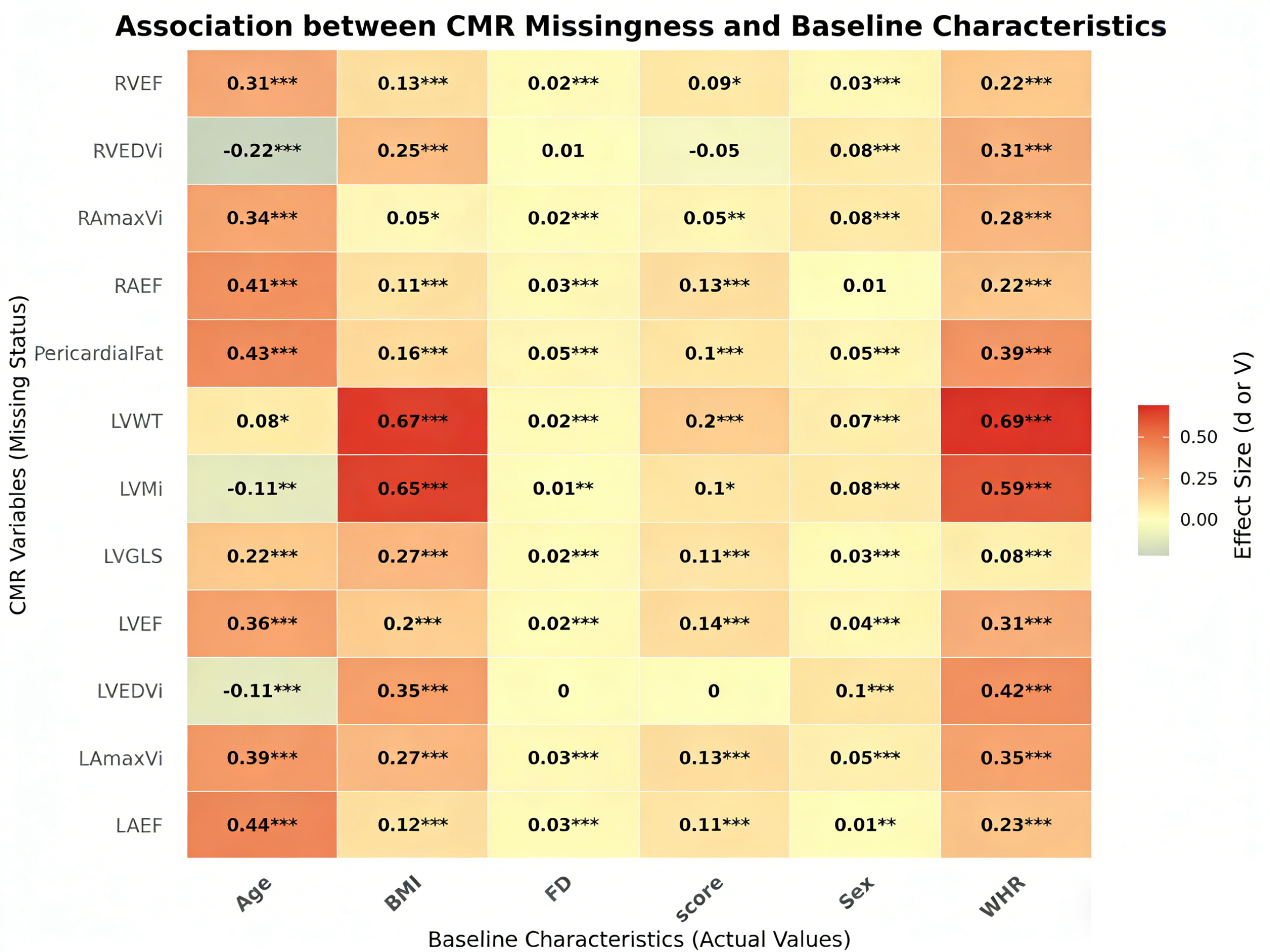
**

Assessment of the correlation between missing CMR Parameter and baseline characteristics. BMI：Body mass index; FD: Frailty Degree WHR: Waist-to-Hip Ratio.

**Supplement Figure 3 Comparison of Effect Estimates: Complete Case Analysis vs. Main Analysis Using Full Sample**


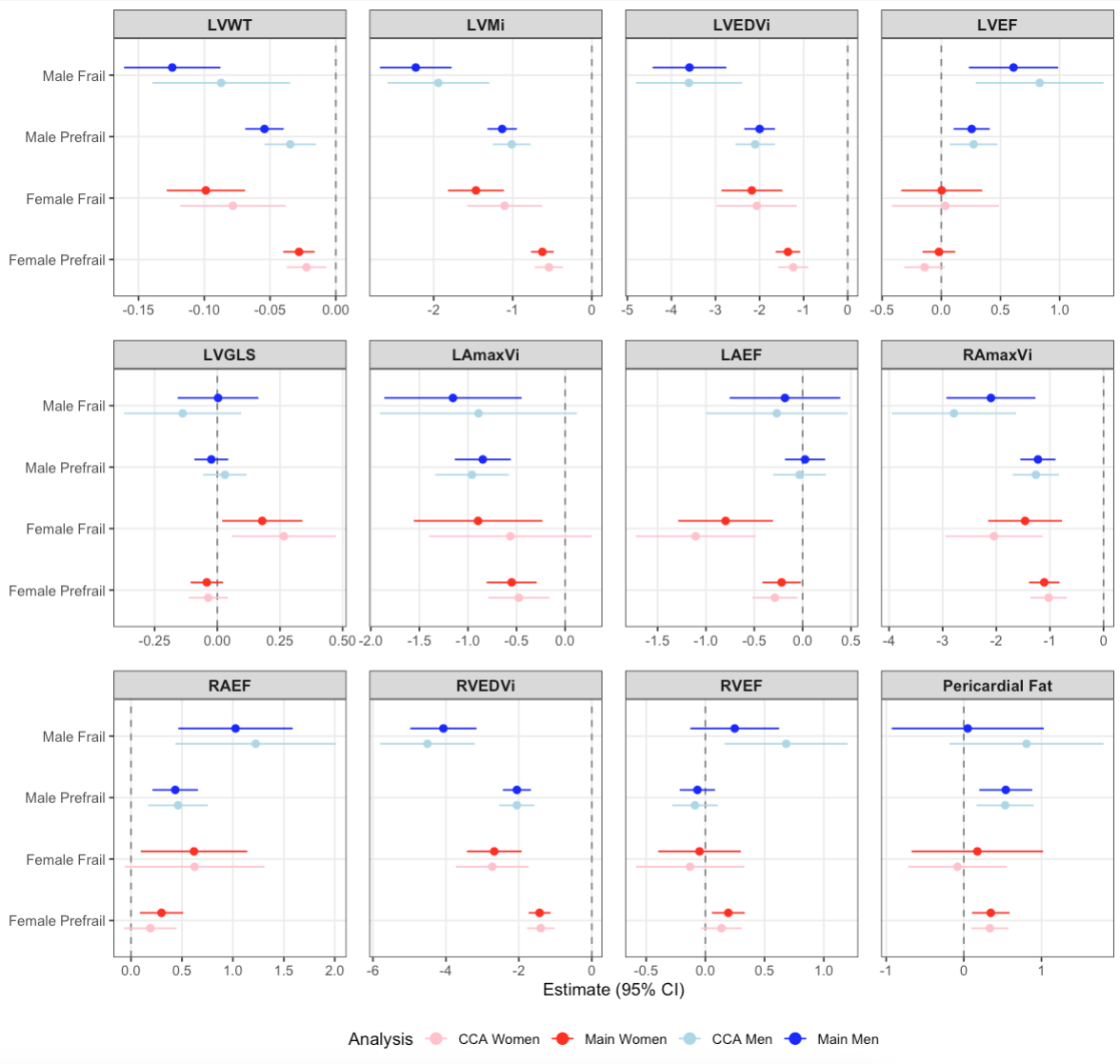


The results of sensitivity analyses comparing effect estimates between complete case analysis (CCA, n = 27,859) and the main analysis using the full sample (n = 48,993), stratified by sex (women and men). The comparison helps assess the robustness of findings to missing data handling.

**Supplement TABLE 4 Sensitivity analysis of the association between frailty status and cardiac parameters under varying delta proportions (δ) in female participants**

| **Parameter** | **Missing rate** | **δ** | **Pre-frail vs Robust** |  | **Frail vs Robust** |  |
| --- | --- | --- | --- | --- | --- | --- |
|  |  |  | β (SE) | p | β (SE) [P] | p |
| **Pericardial Fat** | 33.20% | 0.1 | 0.349 (0.110) | 0.002 | 0.300 (0.317) | 0.36 |
|  |  | 0.2 | 0.322 (0.123) | 0.012 | 0.359 (0.241) | 0.145 |
|  |  | 0.3 | 0.286 (0.119) | 0.019 | 0.279 (0.249) | 0.277 |
| **LVGLS** | 4.70% | 0.1 | -0.042 (0.033) | 0.197 | 0.178 (0.081) | 0.027 |
|  |  | 0.2 | -0.045 (0.033) | 0.173 | 0.165 (0.079) | 0.037 |
|  |  | 0.3 | -0.039 (0.033) | 0.244 | 0.134 (0.089) | 0.14 |
| **LAEF** | 7.30% | 0.1 | -0.214 (0.102) | 0.037 | -0.776 (0.248) | 0.002 |
|  |  | 0.2 | -0.212 (0.099) | 0.032 | -0.784 (0.248) | 0.002 |
|  |  | 0.3 | -0.205 (0.099) | 0.038 | -0.772 (0.238) | 0.001 |
